# It is Good to Recycle: Bringing Megalin Back to the Membrane to Stop Proteinuria

**DOI:** 10.1093/function/zqac056

**Published:** 2022-11-03

**Authors:** Andrew M Hall, Imene Sakhi

**Affiliations:** Institute of Anatomy, University of Zurich, Winterthurerstrasse 190, 8057 Zurich, Switzerland; Department of Nephrology, University Hospital Zurich, Rämistrasse 100, 8091 Zurich, Switzerland; Institute of Anatomy, University of Zurich, Winterthurerstrasse 190, 8057 Zurich, Switzerland

**Keywords:** kidney, proximal tubule, endocytosis, megalin

## A Perspective on “An Adaptable Physiological Model of Endocytic Megalin Trafficking in Ok Cells and Mouse Kidney Proximal Tubule”

### Main

Every medical student knows that proteinuria is an important biomarker of kidney disease. But, how often do we pause to consider what normally stops plasma proteins from ending up in the urine? For sure, it is not the leaky glomerulus, which filters numerous proteins up to the size of albumin, and thus determines their half-life in the circulation. This is the reason why the blood concentration of small proteins such as cystatin c can be used to estimate glomerular filtration rate.^1^ It also means that an efficient retrieval mechanism is required immediately downstream of the glomerulus, and this is a crucial specialized task of the proximal tubule (PT).

The PT scavenges filtered proteins via a process of receptor-mediated endocytosis, involving two large multiligand receptors called megalin and cubilin. Endocytosed proteins are then trafficked quickly to cathepsin-rich lysosomes, where they are degraded to release important nutrients and cargo. The critical importance of this system is demonstrated by the fact that depletion of megalin or cubilin results in large amounts of urinary protein wasting.^2^ Moreover, PT cells display numerous ultrastructural adaptations to performing large amounts of protein uptake and processing, including a highly developed brush border, multiple early endosomes (EEs), and characteristic large apical vacuoles (LAVs), which have hybrid features of sorting and recycling endosomes.^3^ An intricate network of dense apical tubules (DATs) subsequently connects the latter structures to the apical membrane, thus closing the loop. Following binding and internalization, receptors and ligands are quickly dissociated by a process dependent on intense vesicular acidification, driven by the activity of highly expressed V-ATPase pumps.^4^ This allows the former to return back to the cell surface—via the DATs—so the whole procedure can start again. Thus, rapid and continuous receptor recycling is integral to maintaining the high protein retrieval capacity of the PT.

Despite the obvious importance in renal physiology, the kinetics of megalin trafficking in the PT were not well-understood, due to a paucity of realistic in vitro cell models, and the technical challenges of studying this in vivo. When cultured outside their native environment, PT cells rapidly lose endocytotic capacity (which in itself probably tells us something revealing about the influence of the microenvironment in epithelial differentiation). A curious exception are Opossum Kidney (OK) cells, which are somewhat challenging to work with because of the unusual species of origin. Nevertheless, Weisz and colleagues have shown that they maintain certain features of native PT cells—including protein endocytosis—when grown under optimal conditions.^5^

In a new study recently published in *Function*, this group used membrane surface biotinylation assays and antibody staining in OK cells to investigate rates of internalization and steady state distribution of megalin, respectively.^6^ From this, they were subsequently able to generate a new mathematical model of megalin trafficking, allowing them to explore the effects of perturbing individual steps in the pathway. Moreover, by performing antibody staining for established endolysosomal markers in mouse kidney PTs, they have attempted to extrapolate key aspects of their model to the in vivo situation.

**Figure 1.**
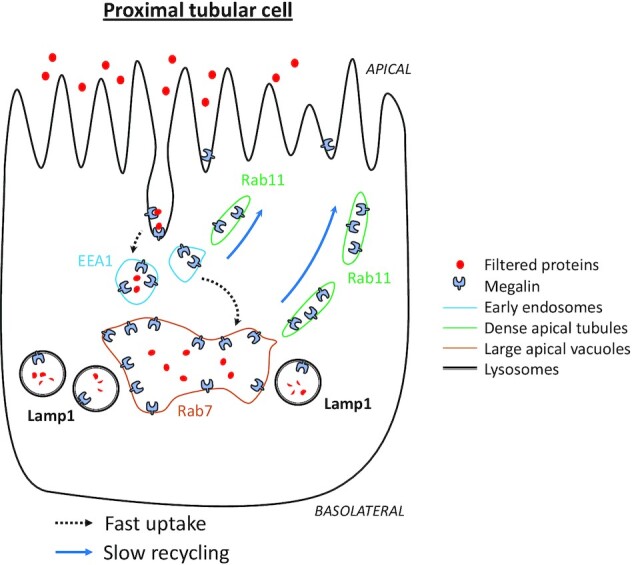
Schematic of megalin trafficking in the PT. The markers used in the study to identify structures are depicted. Filtered plasma proteins bind to megalin and are rapidly internalized by receptor-mediated endocytosis, to prevent wasting in the urine. Dissociation of receptors and ligands occurs in heavily acidified EEs. Proteins are subsequently degraded in cathepsin-rich lysosomes to release nutrients and cargo. Meanwhile, megalin traffics quickly to LAVs, and then more slowly back to the membrane, via Rab11 positive DATs, with only a small amount diverted to lysosomes for destruction. The differential kinetics of trafficking to and from endolysosomes means that most of the megalin pool is intracellular at steady state, and that recycling is the rate-limiting step in determining membrane abundance, and hence protein uptake capacity.

The generation of this model has provided some intriguing and potentially important new insights. For example, rapid internalization explains why—somewhat counter-intuitively—the vast majority of the megalin pool (> 90%) is actually intracellular (Figure 1). Moreover, it seems that megalin transits very quickly through early EEs, meaning that this is not a rate-limiting step in the pathway. Instead, under steady state conditions, most megalin molecules find themselves in LAVs and DATs, en route back toward the membrane via the “slow” recycling pathway (as opposed to “fast” recycling direct from the EEs), with only a tiny fraction heading to lysosomes for imminent retirement (Figure 1). Thus, in this paradigm, it is the recycling kinetics of megalin that critically determine surface abundance, and consequently protein uptake capacity. Meanwhile, from a molecular perspective, spatial staining patterns strongly suggest that the small GTPase Rab11 plays a prominent role in megalin recycling.

Impressive and exciting those these findings are, it is important to consider some potential limitations of the model. For a start, a number of assumptions had to be made—including variables such as megalin synthesis and degradation rates—which are difficult to verify, especially in vivo. Moreover, definitively identifying specific compartments in the PT endolysosomal system is devilishly difficult, due to their close proximity, spatial overlap, and frequent fusion events. Therefore, additional experimental proofs will be required to further validate or refine the working model. However, in the meantime, some of the major conclusions do chime with in vivo functional measurements made recently by our own group using intravital microscopy. For example, we have observed that endocytosed fluorescently labeled proteins transit quickly through EEs into LAVs, implying that megalin does the same.^7^ We also discovered evidence that protein degradation products generated within lysosomes are released into LAVs, and eventually back into the tubular lumen.^7^ Although we cannot be sure of the exact intracellular pathway that they take, it seems likely that the recycling apparatus is involved, thus further highlighting its central importance in PT function.

Assuming that the model of Weisz and colleagues is essentially correct, what might be the wider implications? First, the functional insights help to explain why LAVs and DATs are such prominent structural features in PTs, and further underline the importance of conducting studies with realistic cell models that replicate these. Further work will be required to determine if cubilin also traffics in a similar manner to megalin. Second, a number of genetic disorders affect the PT ELS—such as Dent disease, Lowe syndrome, and cystinosis—causing proteinuria and progressive kidney disease in humans.^8^ Understanding how the movement of megalin is specifically affected in each of these could shed new light on genotype–phenotype relationships. Finally, the findings of this study should prompt a renewed focus on factors that regulate megalin recycling, identification of which could facilitate new strategies to manipulate protein uptake in vivo. These could then potentially be leveraged in certain clinical scenarios; for example, to temporarily prevent megalin-mediated uptake into PTs of nephrotoxins, such as myoglobulin, immunoglobulin light chains, or drugs (eg aminoglycosides).^9,10^

To conclude, these days we are increasingly being reminded of the environmental and health benefits of recycling. According to the work of Weisz and colleagues it seems that the kidney—and in particular the protein-reclaiming PT—got this message a long time ago.

## Funding

A.M.H. is supported by the Swiss National Centre for Competence in Research (NCCR) Kidney Control of Homeostasis and a Swiss National Science Foundation project grant (310030_184688).

## References

[1] Inker LA , Eneanya ND, Coresh J et al. New creatinine- and cystatin C–based equations to estimate GFR without race. N Engl J Med. 2021;385(19):1737–1749. 34554658 10.1056/NEJMoa2102953 PMC8822996

[2] Nielsen R , Christensen EI, Birn H. Megalin and cubilin in proximal tubule protein reabsorption: from experimental models to human disease. Kidney Int. 2016;89(1):58–67. Elsevier B.V.. 26759048 10.1016/j.kint.2015.11.007

[3] Eshbach ML , Weisz OA. Receptor-mediated endocytosis in the proximal tubule. Annu Rev Physiol. 2017;79(10):425–448. Annual Reviews Inc. 27813828 10.1146/annurev-physiol-022516-034234 PMC5512543

[4] Hurtado-Lorenzo A , Skinner M, el Annan J et al. V-ATPase interacts with ARNO and Arf6 in early endosomes and regulates the protein degradative pathway. Nat Cell Biol. 2006;8(2):124–136. 16415858 10.1038/ncb1348

[5] Ren Q , Gliozzi ML, Rittenhouse NL et al. Shear stress and oxygen availability drive differential changes in opossum kidney proximal tubule cell metabolism and endocytosis. Traffic. 2019;20(6):448–459. 30989771 10.1111/tra.12648 PMC7055529

[6] Shipman KE , Long KR, Cowan IA et al. An adaptable physiological model of endocytic megalin trafficking in OK cells and mouse kidney proximal tubule. Function. 2022;3(6):zqac046. 36325513 10.1093/function/zqac046 PMC9614980

[7] Polesel M , Kaminska M, Haenni D et al. Spatiotemporal organisation of protein processing in the kidney. Nat Commun. 2022;13(1):1–13. 36175561 10.1038/s41467-022-33469-5 PMC9522658

[8] van der Wijst J , Belge H, Bindels RJM, Devuyst O. Learning physiology from inherited kidney disorders. Physiol Rev. 2019;99(3):1575–1653. 31215303 10.1152/physrev.00008.2018

[9] Hori Y , Aoki N, Kuwahara S et al. Megalin blockade with cilastatin suppresses drug-induced nephrotoxicity. J Am Soc Nephrol. 2017;28(6):1783–1791. 28052987 10.1681/ASN.2016060606 PMC5461786

[10] Matsushita K , Mori K, Saritas T et al. Cilastatin ameliorates rhabdomyolysis-induced AKI in mice. J Am Soc Nephrol. 2021;32(10):2579–2594. 34341182 10.1681/ASN.2020030263 PMC8722809

